# Quantitative evaluation of longitudinal strain in layer-specific myocardium during normal pregnancy in China

**DOI:** 10.1186/s12947-016-0089-9

**Published:** 2016-11-10

**Authors:** Juan Cong, Zhibin Wang, Hong Jin, Wugang Wang, Kun Gong, Yuanyuan Meng, Yong Lee

**Affiliations:** 1 Department of Echocardiography, The Affiliated Hospital of Qingdao University, Qingdao, Shandong Province China; 2 Department of Cardiology, The Affiliated Hospital of Shandong Medical College, Linyi, Shandong Province China

**Keywords:** Pregnancy, Myocardium, Layer, Strain, Speckle-tracking

## Abstract

**Background:**

The myocardial wall of the left ventricle is a complex, multilayered structure and is not homogenous. The aim of this study was to determine longitudinal strain (LS) in the three myocardial layers in normal pregnant women according to gestation proceedings.

**Methods:**

The advanced two-dimensional speckle tracking echocardiography (2D STE) was performed on 62 women during each pregnancy trimester and 6 to 9 weeks after delivery, while 30 age-matched, healthy, nonpregnant women served as controls. LS on endocardial, mid-myocardial and epicardial layers at 18 cardiac segments were measured.

**Results:**

As gestation proceeded, all of layer-specific LS and global LS progressively decreased, which subsequently recovered postpartum (*P* < 0.05), and the LS gradient between inner and outer myocardium became greater, which reached its maximum in the late pregnancy. Peak systolic LS was the highest at endocardium and the lowest at epicardium, while the highest at the apical level and the lowest at the base (*P* < 0.05). In the early pregnancy and postpartum, LS at basal level was homogenous, meanwhile layer-specific LS showed significant differences at mid-ventricular and apical level throughout the progress of normal pregnancy (*P* < 0.05).

**Conclusions:**

Using 2D STE, three-layer assessment of LS can be performed in pregnant women and shall give us new insights into the quantitative analysis of global and regional LV function during pregnancy. Future studies on the detection of pregnancy related heart disease would require these parameters as reference values for each time point of a normal pregnancy.

## Background

The cardiovascular system undergoes a number of changes during normal pregnancy, including increase in cardiac output and extracellular fluid volume as well as decrease in blood pressure, which are necessary for proceeding of a successful pregnancy. However, dramatical expansion in blood volume during gestation not only can meet the increased metabolic demands of tissue but also can exacerbate cardiac conditions of pregnant women. Although maternal heart disease just complicates a small number of pregnancies overall, it is the leading cause of nonobstetric mortality during pregnancy [1]. So the comprehensive understanding of maternal cardiac function during non-complicated pregnancy is essential to the recognition of cardiac pathology and appropriate monitoring obstetrical patients.

Reduction of myocardial deformation accompanied with progressive cardiac hypertrophy according to gestation period has been described in previous studies [2–4]. These reports focus on the global myocardial wall thickness rather than different layers of the myocardium ranging from endocardium to epicardium. However, the myocardial wall of the left ventricle (LV) is a complex, multilayered structure and is not homogenous [5]. Until recently, speckle tracking echocardiography (STE) has been upgraded, which allows to quantify myocardial function in three layers [6]. Layer-specific differences of myocardial performance and deformation have been analyzed in normal subjects and different heart diseases [6–9]. However, still little is known about how differently multilayer myocardium contributes to myocardial deformation throughout the progress of normal pregnancy. In this study, we used the advanced two-dimensional (2D) STE to evaluate myocardial deformation within each of three myocardial layers, an endocardial, mid-myocardial, and epicardial layer. The aim of this study was to determine longitudinal strain (LS) in each of the three myocardial layers in normal pregnant women according to gestation proceeding.

## Methods

Sixty-two of 71 subjects with singleton pregnancy (mean age, 28.2 ± 6.6 years; range, 24–36 years) and 30 age-matched, healthy, nonpregnant women were involved after informed consent and with approval from the Affiliated Hospital of Qingdao University Ethics Committee. Four visits were planned during the study: trimester 1, 12–14 weeks; trimester 2, 22–28 weeks; trimester 3, 36–40 weeks and 6–9 weeks after delivery. Enrolled criteria of healthy pregnant women was that they were without medical diseases, such as cardiovascular disorders, renal disease etc., and without obstetrical complications, such as gestational diabetes mellitus or pregnancy-induced hypertension. Subjects who had poor echo quality or any fetal abnormalities were excluded from the study.

Subjects underwent standard 2D echocardiographic examinations using commercially available ultrasound machine (Vivid E9; GE Healthcare, Horten, Norway) equipped with a M5S transducer. The following parameters were performed by M-Mode in the parasternal long-axis view as recommended [10]: interventricular septum (IVSd), posterior wall (PWd), left ventricular end-diastolic (LVEDd) and endsystolic (LVEDs) diameters. LV ejection fraction and stroke volume were calculated as previously described. Relative wall thickness (RWT) was calculated as (IVSd + PWd)/LVEDd. Cardiac indices were normalized for body surface area. Three apical long-axis scans were obtained at the apical four-chamber, two-chamber, and long-axis planes. Moreover, standard short-axis views were acquired at the basal, mid-ventricular and apical level. Tissue pulsed Doppler was recorded in the apical four- and two- chamber view. The average of peak systolic velocities (Sm), early diastolic velocities (Em), and late diastolic velocities (Am) at the septal, lateral, anterior, and inferior at mitral annulus were computed. The LV was divided into 18 cardiac segments: 6 segments (anterior, anteroseptal, inferior, lateral, posterior, and septal) at 3 levels (basal, mid, and apical). The frame rate was 52–94 frames/s. Peak systolic LS was obtained in three myocardial layers from the apical views. All image acquisitions were performed throughout three consecutive cardiac cycles during breath-holds.

**Fig 1:**
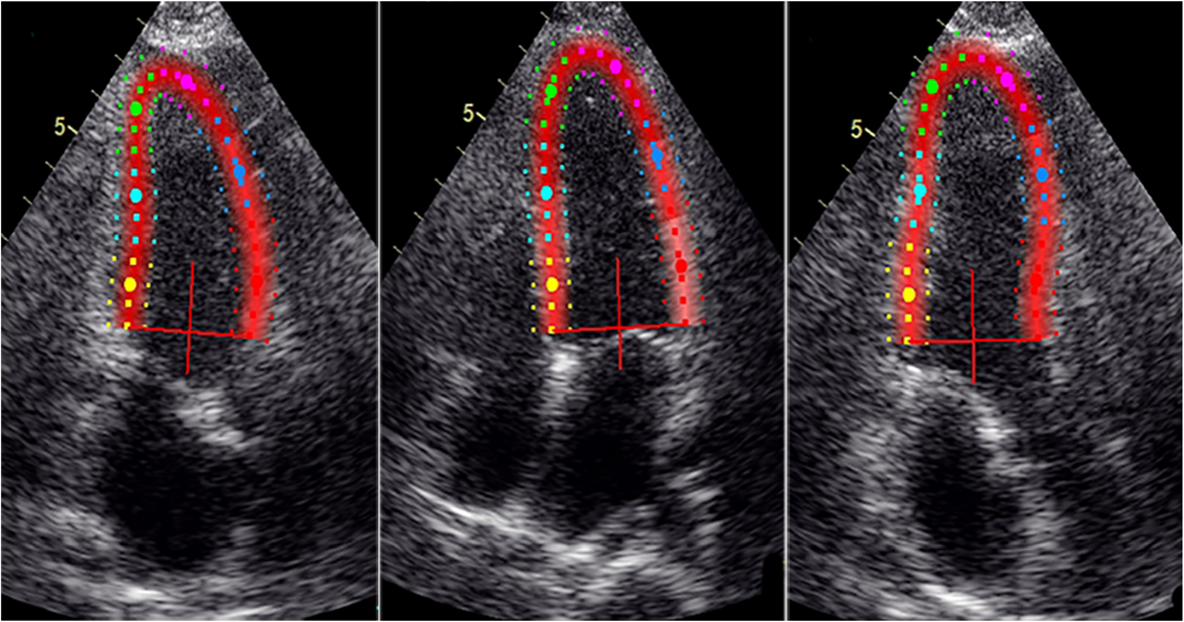
Multiple-dotted lines in three myocardial layers at the three parasternal long-axis scans. The endocardial borders are delineated in the end-systolic frame of the images at the apical four-chamber, two-chamber, and long-axis planes. Subsequently, the myocardial wall is automatically defined with multiple chains of nodes for allowing assessment of longitudinal endocardial, mid-myocardial and epicardial strains

**Fig 2:**
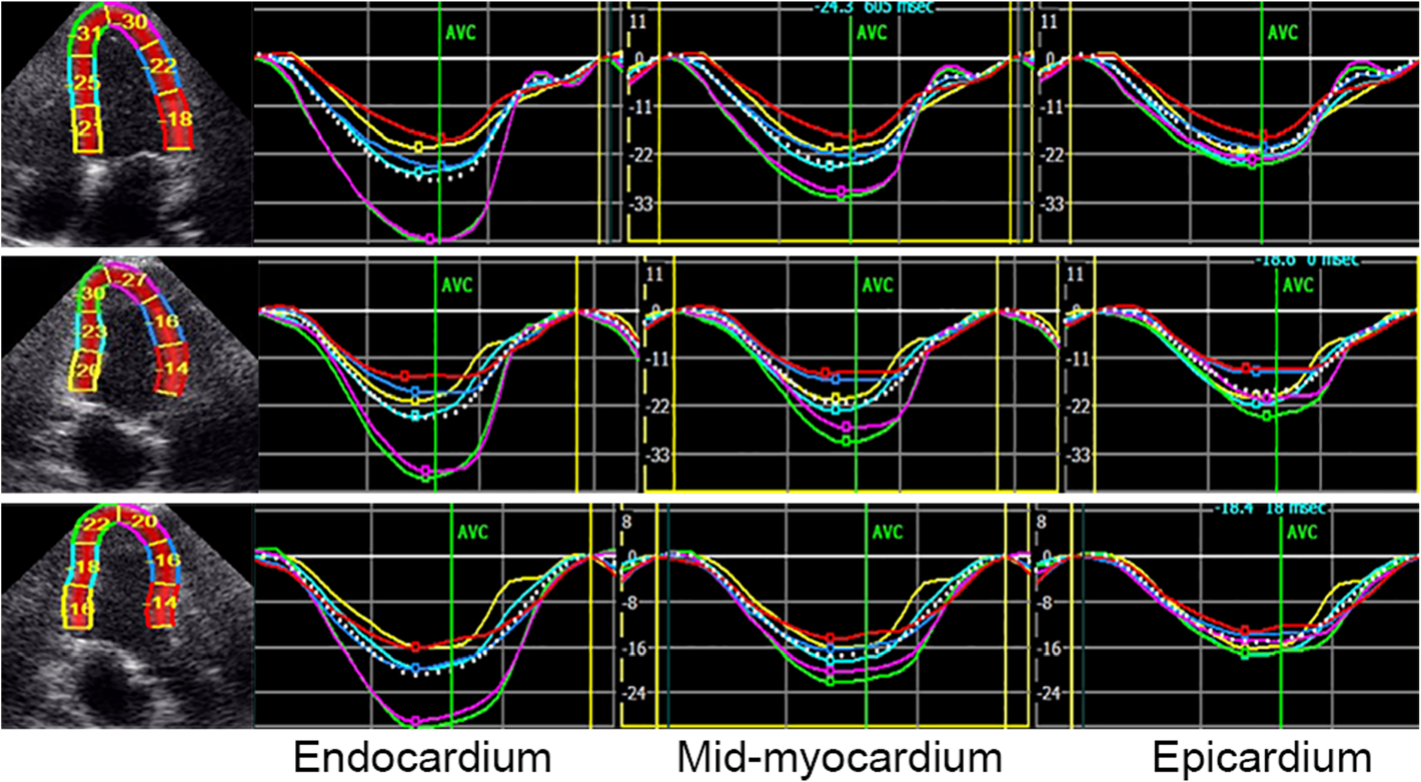
Layer-specific strain curves in each segment. Quantitative myocardial parameters for each segment are evaluated in an 18 segment LV model (six segments at each level) at all three acquired parasternal long-axis views

All grayscale images of the apical long-axis 2D echocardiography were analyzed frame by frame using an offline software package (EchoPAC, PC version 113.1). The endocardial borders were delineated in the end-systolic frame of the images at the 3 apical views. Subsequently, the myocardial wall was automatically defined with multiple chains of nodes for allowing assessment of longitudinal endocardial, mid-myocardial and epicardial strains (Fig. 1). Then, quantitative myocardial parameters for each segment were evaluated in an 18 segment LV model (six segments at each level) at all three acquired apical long-axis views (Fig. 2). Deformation parameters were determined as average of the three consecutive beats. The myocardial deformation at the basal, mid-ventricular and apical levels were averaged to global longitudinal strain (GLS) in the endocardial layer (GLS-endo), in the mid-myocardial layer (GLS-mid) and in the epicardial layer (GLS-epi), respectively. All segmental values were averaged to ventricular GLS.

Statistical analysis was performed with SPSS (version 17.0). Data were shown as mean ± SD. LS was presented in its absolute value. Comparison of continuous variables was performed with independent sample *t* tests or ANOVA as appropriate. Reproducibility was assessed by the mean percentage error (absolute difference divided by the mean of the 2 observations). *P* < 0.05 was considered to indicate statistical significance.

## Results

Table 1 shows the clinical and hemodynamic characteristics of the pregnant women and controls. As the pregnancy progressed, diastolic blood pressure and mean blood pressure were slightly reduced but followed by a mild increase toward the third trimester. Due to late increase in heart rate and in stroke volume, the cardiac index increased progressively by a mean of 33 % between the first and third trimesters.

**Table 1:** Clinical and hemodynamic characteristics in pregnant women

Variable	Controls	Trimester 1	Trimester 2	Trimester 3	Postpartum
	-	(13.6 ± 2.2)wk	(24.8 ± 3.4)wk	(38.1 ± 2.6)wk	(7.4 ± 2.4)wk
No. participants	30	62	62	62	62
Weight(kg)	59.3 ± 11.4	61.1 ± 9.6	68.2 ± 8.5*^†‡§^	72.8 ± 7.0*^†^	62.8 ± 13.5^‡^
BSA(m^2^)	1.63 ± 0.12	1.64 ± 0.20	1.66 ± 0.11^‡^	1.74 ± 0.10*^†^	1.66 ± 0.18^‡^
Heart rate(bpm)	81.1 ± 14.3	82.6 ± 12.4	85.1 ± 17.2	90.1 ± 9.8*^†^	81.3 ± 14.2^‡^
SBP(mmHg)	104.2 ± 10.7	105.1 ± 8.7	102.9 ± 11.5	107.6 ± 9.6	110.2 ± 10.3
DBP(mmHg)	64.4 ± 8.0	64.3 ± 6.9	61.3 ± 6.8^‡§^	67.2 ± 7.9	68.6 ± 6.2
MBP(mmHg)	79.3 ± 8.5	80.5 ± 7.0	76.5 ± 6.2^‡§^	81.7 ± 7.7	83.3 ± 8.1
CI (l · min^-1^ · m^-2^)	2.98 ± 0.76	3.01 ± 0.66	3.59 ± 0.79*	4.06 ± 0.72*^†^	3.36 ± 0.72^‡^
SVI(ml/m^2^)	34.58 ± 6.23	35.11 ± 9.60	37.56 ± 8.52	39.03 ± 5.34*^†^	36.56 ± 7.54

Data are given as mean ± SD, BSA indicates Body surface area

*SBP* systolic blood pressure, *DBP* diastolic blood pressure, *MBP* mean blood pressure, *CI* cardiac index, *SVI* stroke volume index

*P < 0.05 vs. Controls; ^†^ *P* < 0.05 vs. Trimester 1; ^‡^ *P* < 0.05 vs. Trimester 3; ^§^ *P* < 0.05 vs. Postpartum

Table 2 summarizes the parameters of LV geometry and function in the study population. There was a progressive increase in LV volume and LV wall thickness, which resulted in slightly eccentric hypertrophy during pregnancy. From the first trimester to the third trimester, ejective fraction and peak myocardial velocity of mitral annulus Sm decreased by 4.44 and 8.37 %, respectively. Those changes had almost returned to control levels in the postpartum study.

**Table 2:** The morphological and functional changes in the left ventricle in pregnant women

Variable	Controls	Trimester 1	Trimester 2	Trimester 3	Postpartum
	-	(13.6 ± 2.2)wk	(24.8 ± 3.4)wk	(38.1 ± 2.6)wk	(7.4 ± 2.4)wk
LVEDd (mm)	44.18 ± 2.50	45.28 ± 2.83	46.88 ± 3.40	48.84 ± 3.26*^†^	46.31 ± 3.14
LVEDs (mm)	28.08 ± 2.84	28.24 ± 3.21	29.62 ± 2.91	30.46 ± 2.81*	28.72 ± 2.51
RWT	0.25 ± 0.09	0.27 ± 0.07	0.28 ± 0.05	0.29 ± 0.06*	0.26 ± 0.06
LVMi (g/m^2^)	59.25 ± 18.00	62.57 ± 24.81	66.37 ± 16.74	71.23 ± 14.46*^†^	63.14 ± 16.08^‡^
Sphericity index	0.27 ± 0.04	0.31 ± 0.07*	0.32 ± 0.05*^§^	0.32 ± 0.06*	0.28 ± 0.06^‡^
LVEDV(ml)	76.14 ± 21.20	81.39 ± 19.54	83.78 ± 14.58	87.72 ± 17.18*^†^	82.77 ± 16.85^‡^
LVESV(ml)	32.00 ± 7.29	33.91 ± 6.16	36.30 ± 7.96*	39.67 ± 7.98*	34.08 ± 8.32
LVEDV index (ml/m^2^)	44.06 ± 17.44	41.93 ± 18.61	49.33 ± 14.49^†^	51.67 ± 12.65*^†^	46.63 ± 16.49
EF (%)	67.57 ± 5.12	68.00 ± 5.21	65.46 ± 4.49^†^	64.98 ± 3.93*^†^	66.89 ± 4.91
Sm (cm/min)	7.01 ± 1.63	7.06 ± 1.24	6.82 ± 1.03	6.47 ± 1.21*^†^	6.93 ± 1.72
E/Em	8.33 ± 1.07	11.56 ± 2.08*	12.46 ± 3.18*	13.34 ± 4.33*	9.04 ± 4.05

Data are given as mean ± SD, LVEDd indicates left ventricular end-diastolic dimension

*LVEDs* left ventricular end-systolic dimension, *RWT* relative wall thickness, *LVMi* left ventricular mass index, *LVEDV* indicates left ventricular end-diastolic volume, *LVESV* left ventricular end-systolic volume, *EF* ejection fraction, *Sm* average of peak systolic velocities, *E* peak early diastole transmitral wave velocity, *Em* average of peak early diastolic velocities

*P < 0.05 vs. Controls; ^†^ *P* < 0.05 vs. Trimester 1; ^‡^ *P* < 0.05 vs. Trimester 3;^§^ *P* < 0.05 vs. Postpartum

**Fig 3:**
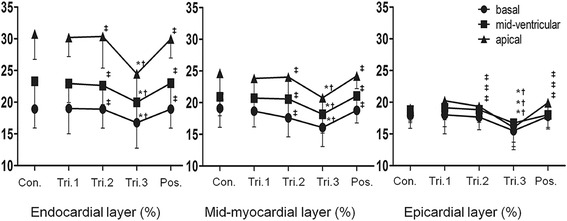
Evolution of three-layer longitudinal strain during pregnancy. Data are presented as mean ± SD in absolute values. *P < 0.05 vs. Controls; ^†^ *P* < 0.05 vs. Trimester 1; ^‡^ *P* < 0.05 vs. Trimester 3; ^§^ *P* < 0.05 vs. Postpartum

Data of layer-specific GLS in pregnant women is depicted in Table 3. Among 4464 segments with pregnant women, 4132 (92.56 %) segments were successfully analyzed while 512 (94.81 %) out of 540 segments with control subjects were assessed by modified speckle-tracking imaging. All of GLS showed significant decrease in late pregnancy, consistent with the slightly decline of ejective fraction, and followed by a recovery postpartum. During normal pregnancy, GLS was the highest at endocardium, lower at mid-myocardium, and the lowest at epicardium and the deformation of all three-layer myocardial wall showed a decreasing tendency in the third trimester, which subsequently recovered after delivery (Fig. 3). As the gestation proceeded, the absolute difference in GLS-endo and GLS-epi became greater, which reached its maximum in the late pregnancy. The global epicardial-to-endocardial gradient was 21.21 % in trimester 1, 22.37 % in trimester 2, 24.81 % in trimester 3, and 20.40 % postpartum, respectively.

**Table 3:** Three-layer longitudinal strain evolution during pregnancy

	Controls	Trimester 1	Trimester 2	Trimester 3	Postpartum
		(13.6 ± 2.2)wk	(24.8 ± 4.4)wk	(38.1 ± 2.6)wk	(7.4 ± 2.4)mo
GLS-endo (%)	24.32 ± 4.11	24.10 ± 2.78	23.97 ± 2.98^‡^	21.40 ± 3.07*^†^	24.02 ± 3.17^‡^
GLS-mid (%)	21.15 ± 2.90	20.98 ± 3.20	21.03 ± 3.21^‡^	18.34 ± 2.76*^†^	21.18 ± 2.26^‡^
GLS-epi (%)	18.96 ± 2.84	19.01 ± 3.33	18.61 ± 2.83^‡^	16.09 ± 2.57*^†^	19.12 ± 3.33^‡^
Avg.GLS (%)	20.34 ± 2.97	21.09 ± 3.15	21.21 ± 2.60^‡^	18.62 ± 2.81*^†^	20.95 ± 3.06^‡^

Data are given as mean ± SD in absolute values. GLS indicates the global longitudinal strain; GLS-endo, the average value of global longitudinal strain in the endocardial layer at the basal, mid-ventricular and apical levels; GLS-mid, the average value of global longitudinal strain in the mid-myocardial layer at the basal, mid-ventricular and apical levels; GLS-epi, the average value of global longitudinal strain in the epicardial layer at the basal, mid-ventricular and apical levels

*P < 0.05 vs. Controls; ^†^ *P* < 0.05 vs. Trimester 1; ^‡^ *P* < 0.05 vs. Trimester 3; ^§^ *P* < 0.05 vs. Postpartum

**Fig 4:**
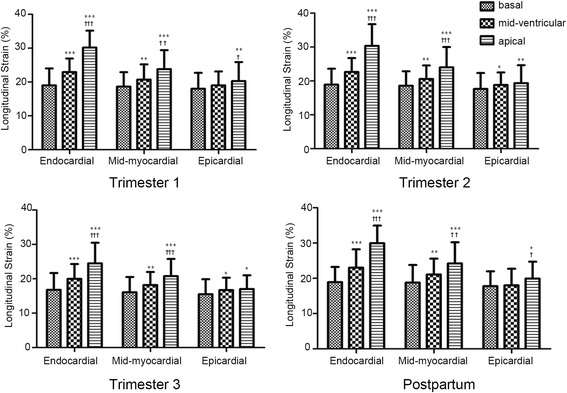
LV longitudinal strain of endocardial, mid-myocardial, and epicardial layers in normal pregnancy. Data are presented as mean ± SD in absolute values. *P < 0.05 vs. basal; **P < 0.01 vs. basal; ***P < 0.001 vs. basal; ^†^ *P* < 0.05 vs. mid-ventircular; ^† †^ *P* < 0.01 vs. mid-ventircular; ^† † †^ *P* < 0.001 vs. mid-ventircular

The peak LS of three myocardial layers at the basal, mid-ventricular, and apical levels of the LV in pregnancy are reported in Table 4. Considering a layer-specific analysis of myocardial deformation in normal pregnancy, all of the peak systolic LS in the endocardium, mid-myocardium and epicardium were gradually increased from the base to the apex, the greatest in the apical level and the lowest in the base (Fig. 4). Moreover, the difference between inner and outer myocardium at each level increased during pregnancy. The epicardial-to-endocardial gradient was 5.26, 21.53 and 33.65 % at the basal, mid-ventricular and apical level in the first trimester, 6.66, 22.04 and 36.26 % in the second trimester, 7.51, 22.37 and 38.08 % in the third trimester, respectively, meanwhile it was 5.98, 22.76 and 33.51 % postpartum compared with 3.97, 23.29 and 38.15 % in the control subjects, respectively. In the trimester 1 and the post delivery period, peak systolic LS in the three layers were similar at the basal level. However, at both mid-ventricular and apical level, there was significantly difference among layer-specific myocardial deformation during the gestation period and postpartum.

**Table 4:** Three-layer longitudinal strain at the basal, mid-ventricular, and apical levels of the left ventricle in pregnancy

	Controls	Trimester 1	Trimester 2	Trimester 3	Postpartum
		(13.6 ± 2.2)wk	(24.8 ± 4.4)wk	(38.1 ± 2.6)wk	(7.4 ± 2.4) wk
Basal level (%)					
Endocardial layer (%)	18.62 ± 4.01	19.01 ± 5.67	18.91 ± 4.67^‡^	16.77 ± 4.91*^†^	18.91 ± 4.33^‡^
Mid-myocardial layer (%)	19.09 ± 4.61	18.65 ± 4.27	18.59 ± 4.67^‡^	16.08 ± 4.57*^†^	18.79 ± 5.07^‡^
Epicardial layer (%)	17.88 ± 3.72	18.01 ± 4.70	17.65 ± 4.70^‡^	15.51 ± 4.36*^†^	17.78 ± 4.22^‡^
*P* -value (between layers)	. > 0.05	>0.05	<0.05	< 0.05	> 0.05
Mid-ventricular level (%)					
Endocardial layer (%)	23.31 ± 5.16	22.95 ± 4.53	22.64 ± 4.11^‡^	19.98 ± 3.31*^†^	23.02 ± 5.26^‡^
Mid-myocardial layer (%)	20.92 ± 5.22	20.72 ± 4.31	20.57 ± 4.29^‡^	18.18 ± 3.80*^†^	21.07 ± 4.92^‡^
Epicardial layer (%)	17.88 ± 3.72	18.01 ± 4.70	17.65 ± 4.70^‡^	15.51 ± 4.36*^†^	17.78 ± 4.22^‡^
*P*-value (between layers)	< 0.01	< 0.01	< 0.001	< 0.001	< 0.01
Apical level (%)					
Endocardial layer (%)	30.77 ± 6.23	30.19 ± 5.65	30.36 ± 7.44^‡^	27.47 ± 6.97*^†^	29.96 ± 5.44^‡^
Mid-myocardial layer (%)	24.63 ± 6.05	23.83 ± 5.19	24.03 ± 6.06^‡^	20.77 ± 5.47*^†^	24.21 ± 6.46^‡^
Epicardial layer (%)	19.03 ± 4.77	20.03 ± 5.65	19.35 ± 5.30^‡^	17.01 ± 4.79*^†^	19.92 ± 4.82^‡^
*P*-value (between layers)	< 0.001	< 0.001	< 0.001	< 0.001	< 0.001

Data are given as mean ± SD in absolute values; *P < 0.05 vs. Controls; ^†^ *P* < 0.05 vs. Trimester 1; ^‡^ *P* < 0.05 vs. Trimester 3

Table 5 summarizes the univariate relations of layer-specific strain in the third trimester during pregnancy. The GLS in the endocardial, mid-myocardial and epicardial layers showed significant associations with gestation period, maternal age, BSA and with other ventricular parameters as LVEDd, RWT, sphericity index as well as LVEF.

**Table 5:** Univariate relations (*r* coefficient and significance) of layer-specific strain components in the third trimester during pregnancy

Variable	Avg.GLS (*P*-value)	GLS-endo (*P*-value)	GLS-mid (*P*-value)	GLS-epi (*P*-value)
Gestation period(w)	0.385(*P* < 0.01)	0.290(*P* < 0.05)	0.472(*P* < 0.01)	0.487(*P* < 0.01)
Age(year)	0.368(*P* < 0.01)	0.291(*P* < 0.01)	0.372(*P* < 0.01)	0.473(*P* < 0.01)
BSA(m^2^)	0.510(*P* < 0.01)	0.610(*P* < 0.01)	0.479(*P* < 0.01)	0.388(*P* < 0.01)
LVEDd(mm)	0.275(*P* < 0.05)	0.223(*P* < 0.05)	0.263(*P* < 0.05)	0.337(*P* < 0.01)
RWT	0.308(*P* < 0.01)	0.284(*P* < 0.05)	0.342(*P* < 0.01)	0.281(*P* < 0.05)
Sphericity index	0.265(*P* < 0.05)	0.297(*P* < 0.01)	0.232(*P* < 0.05)	0.244(*P* < 0.05)
EF(%)	0.686(*P* < 0.01)	0.640(*P* < 0.01)	0.682(*P* < 0.01)	0.707(*P* < 0.01)

Abbreviations as in Tables 1, 2, and 3. Values of GLS, GCS, GRS and GAS considered as ‘positive’ (sign +) to build the univariate relations in order to homogenize the results of analyses and strengthen their clinical meaning: the higher the values, the better is the strain deformation independent of the plus/minus sign

For deformational assessments of the endocardial, mid-myocardial, and epicardial layers, intraobserver and interobserver variability were 11.7 %, 12.3 %, 12.4 %, respectively, and 12.3 %, 12.8 %, 12.6 %, respectively.

## Discussion

A comprehensive evaluation of systolic LV function requires the consideration of all of the parameters on the global and local level. The indices of LV myocardial strain describe ventricular deformation at regional level, which have been demonstrated to be more sensitive and accurate in the identification of LV functional impairment compared with global performance of the heart [11, 12]. A number of studies on maternal myocardial deformation during pregnancy have been published, but there is still controversy regarding the changes in LV performance [2–4, 13, 14]. Moreover, these findings analyzed the myocardial function considering the complete wall thickness without further distinction between different layers of the myocardium. To the best of our knowledge, this study is the first to demonstrate the deformation of layer-specific myocardium, endocardial, mid-ventricular and epicardial layers during normal pregnancy using the modified 2D STE.

During pregnancy, a series of dramatic changes in cardiovascular system, including increases in blood volume and cardiac output as well as decreases in LV afterload, occur to meet the drastic increases in the metabolic demands and thus supply adequate blood to the growth of the fetus. In the present study, LV size gradually enlarged according to gestational weeks. From early to late pregnancy, cardiac index progressively elevated by about one third resulting from accelerated heart rate and increased stroke volume. Blood pressure, afterload of LV, slightly decreased during mid-pregnancy and tended to increase toward the third trimester. As a response to the changes in the volume and pressure, a slight cardiac hypertrophy occurred to enable the heart to fulfill its function during pregnancy. These changes slowly returned to normal value postpartum. Our data about hemodynamic and morphological changes during pregnancy is in concordance with the previous studies [2–4, 15].

The quantitative assessment of LS is an important part of echocardiophic analysis. The longitudinal behavior of ventricular wall is principal deformation of the heart [11], which represents as shortening and lengthening of myocardial fibers from the base to the apex [16]. In the current study, we found the ventricular GLS decreased significantly in late pregnancy. This is similar to the previous studies, which had demonstrated that the global myocardial deformation in three dimensions, longitudinal, circumferential as well as radial strain, reduced markedly in the third trimester [2, 3]. Those reports considered multilayered structure of the ventricular wall as a total thickness. However, the LV wall is not homogenous, which is composed of three layers of myocardium ranging from endocardium to epicardium. Spatial configuration of ventricular myocardial fibers in the subendocardial and subepicardial layers provide sequential contractile activity of ventricle and contribute to the equal redistribution of stress and strain of heart [5, 17]. Furthermore, histologic analyses have proved that different diseases could injury the myocardial layers to a different extent and could result in alternated predominant dysfunction in specific layers [18, 19]. Thus, evaluation myocardial deformation just across the ventricular wall thickness is not able to provide comprehensive information of the cardiac function.

Our data showed that, with the pregnancy proceeding, all GLS in each of three myocardial layers, endocardium, mid-myocardium, and epicardium reduced and reached its lowest in the trimester 3, which recovered after delivery. An increased epicardial-to-endocardial gradient of LS were described from the basal to the apical level of the ventricle in pregnant women, which is in agreement with the findings of layer-specific LS in normal non-pregnant subjects [7, 9]. We noticed that the deformation of three-layer myocardium at the basal level was significantly different in the second and the third trimester, while it was almost homogenous in the early pregnancy and postpartum as well as non-pregnant controls. This finding may be resulted from hemodynamic and cardiac morphological changes in the middle and late pregnancy.

Both ventricular GLS and layer-specific LS reflect myocardial function regulated by regional condition of ventricular loading and chamber morphology. To produce the same stroke volume, there is an inverse relationship between heart size and strain [20]. During early and middle pregnancy, a decreased cardiac afterload was balanced by an enlarged ventricular chamber and thus stroke volume index does not significantly increase. As a result, global and layer-specific strain keeps stable. Hypertrophy is commonly seen as a primary mechanism of the heart to reduce stress on the ventricular walls [21]. At the end of pregnancy, a mild cardiac hypertrophy happened, but it could not completely balance further increase in LV size and higher cardiac afterload. Therefore, longitudinal deformation both across total wall thickness and in multiple-layer myocardium significantly decreased despite an increasing stroke work.

In addition, LV hypertrophy during pregnancy is a complex process including a number of changes in extracellular matrix [2], the hormonal levels as well as the molecular mechanism. Recent studies have shown there is a unique system of molecular signaling pathway involved in pregnancy-induced hypertrophy [22–24]. The ubiquitin-proteasome system is known to play an important role in the degradation of damaged and misfolded proteins in the heart [25]. Both trypsin-like activity (β2) and chymotrypsin-like activity (β5), the subunit of the ubiquitin-proteasome system, were reported significantly elevated to the highest level in the subendocardium in a canine model of LV hypertrophy [26]. This possibly is one of the reasons for different strains in multiple myocardium during pregnancy. However, the precise role of molecular mechanism in physiological heart hypertrophy during pregnancy is still little known yet.

The present study on three-layer myocardial deformation in normal pregnancy can be essential for better understanding of different pathological or disease stage during pregnancy, and this is likely to give new insight into the clinically relevant therapies for pregnancy-induced cardiovascular complications, such as peripartum cardiomyopathy and pre-eclampsia.

The limitations of the present study are as follows. Firstly, the vendor-specific software to analyze layer-specific strain has not been validated by sonomicrometry. However, the current speckle tracking software has already been proved to be in coincidence with MRI both experimentally and clinically for detailed evaluation of layer-specific myocardial function. Secondly, due to continuity of myocardial fibers, the deformation parameters within the three layers are not completely isolated and absolute, and they influence each other. Thirdly, STE analysis depends on spatial resolution that tends to decrease with depth settings and higher frame rates. Fourthly, there are several dimensional movements of LV myocardium, longitudinal, circumferential and radial axis. The present study just focuses on the longitudinal deformation, the primary strain of the heart during pregnancy. Lastly, it doesn’t have clear clinical implications of layer-specific LS, since at the moment it’s hard to see a real utility in measuring a well-known physiological effect in normal pregnancies.

## Conclusions

Using 2D STE, three-layer assessment of LS can be performed in pregnant women and shall give us new insights into the quantitative analysis of global and regional LV function during the pregnancy. Future studies on the detection of pregnancy related heart disease would require these parameters as reference values for each time point of a normal pregnancy.

## Abbreviations

2D: Two-dimensional
Am: The average of late diastolic velocities at mitral annulus
Em: The average of early diastolic velocities at mitral annulus
GLS: Global longitudinal strain
GLS-endo: Global longitudinal strain in the endocardial layer
GLS-epi: Global longitudinal strain in the epicardial layer
GLS-mid: Global longitudinal strain in the mid-myocardial layer
IVSd: Interventricular septum diameter
LS: Longitudinal strain
LV: Left ventricle
LVEDd: Left ventricular end-diastolic diameter
PWd: Posterior wall diameter
RWT: Relative wall thickness
Sm: The average of peak systolic velocities at mitral annulus
STE: Speckle tracking echocardiography.
